# Mechanistic platform knowledge of concomitant sugar uptake in *Escherichia coli* BL21(DE3) strains

**DOI:** 10.1038/srep45072

**Published:** 2017-03-23

**Authors:** David J. Wurm, Johanna Hausjell, Sophia Ulonska, Christoph Herwig, Oliver Spadiut

**Affiliations:** 1 Research Division Biochemical Engineering, Institute of Chemical Engineering, Vienna University of Technology, Vienna, Austria; 2 Christian Doppler Laboratory for Mechanistic and Physiological Methods for Improved Bioprocesses, Institute of Chemical Engineering, Vienna University of Technology, Vienna, Austria

## Abstract

When producing recombinant proteins, the use of *Escherichia coli* strain BL21(DE3) in combination with the T7-based pET-expression system is often the method of choice. In a recent study we introduced a mechanistic model describing the correlation of the specific glucose uptake rate (q_s,glu_) and the corresponding maximum specific lactose uptake rate (q_s,lac,max_) for a pET-based *E. coli* BL21(DE3) strain producing a single chain variable fragment (scFv). We showed the effect of q_s,lac,max_ on productivity and product location underlining its importance for recombinant protein production. In the present study we investigated the mechanistic q_s,glu_/q_s,lac,max_ correlation for four pET-based *E. coli* BL21(DE3) strains producing different recombinant products and thereby proved the mechanistic model to be platform knowledge for *E. coli* BL21(DE3). However, we found that the model parameters strongly depended on the recombinant product. Driven by this observation we tested different dynamic bioprocess strategies to allow a faster investigation of this mechanistic correlation. In fact, we succeeded and propose an experimental strategy comprising only one batch cultivation, one fed-batch cultivation as well as one dynamic experiment, to reliably determine the mechanistic model for q_s,glu_/q_s,lac,max_ and get trustworthy model parameters for pET-based *E. coli* BL21(DE3) strains which are the basis for bioprocess development.

The bacterium *Escherichia coli* is one of the most widely used host organisms for recombinant protein production^1^,^2^,^3^,^4^. It features several advantages including extensive knowledge about its genome coming along with the availability of numerous established methods for genetic modification, multiple engineered strains as well as a dazzling array of expression plasmids^2^,^5^. Amongst those, the T7-based pET expression plasmids are frequently employed since the strong T7 promoter^6^ allows exceptionally high yields of recombinant product^5^,^7^. The most common approach for inducing these pET-based *E. coli* strains is by IPTG^7^,^8^. IPTG is not metabolized by bacteria, which is why one-point addition is sufficient to guarantee induction^5^. However, IPTG is known to put a high metabolic burden on *E. coli*^9^,^10^ and is often associated with the generation of misfolded protein aggregates, called inclusion bodies^11^,^12^. In contrast, the alternative inducer lactose has been shown to favour the production of soluble product and trigger enhanced productivity^13^,^14^,^15^,^16^. Even though lactose metabolism is topic of some recent studies^17^, lactose is scarcely used in biochemical engineering since induction entails the challenge of continuous supply of the disaccharide as it gets metabolized by *E. coli*. Furthermore, cultivations have to be conducted at limiting amounts of glucose as otherwise lactose uptake is inhibited due to the well-known phenomenon of carbon catabolite repression (e.g. refs 18, 19 and 20).

To shed more light on the mechanistic correlation between the uptake of glucose and lactose, we recently performed a comprehensive study with a recombinant pET-based *E. coli* BL21(DE3) strain producing a single chain variable fragment (scFv) against celiac disease^16^. We succeeded in establishing a mechanistic model of the specific glucose uptake rate (q_s,glu_) and the corresponding maximum specific lactose uptake rate (q_s,lac,max_). Furthermore, we showed that q_s,lac,max_ impacted productivity as well as product location and is thus a crucial parameter for recombinant protein production. Finally, we hypothesized that this mechanistic correlation might describe platform knowledge for *E. coli* BL21(DE3) strains carrying the pET expression system and proposed to conduct at least four bioreactor cultivations (batch and fed-batch experiments) to determine the mechanistic model for any pET-based *E. coli* BL21(DE3) strain^16^.

In the present study we put our hypothesis to test and investigated the mechanistic correlation of q_s,glu_ and q_s,lac,max_ for four pET-based *E. coli* BL21(DE3) strains producing different recombinant proteins. We were able to prove the mechanistic model to be applicable and concluded that this model in fact describes platform knowledge for pET-based *E. coli* BL21(DE3) strains. However, we found that model parameters were strongly dependent on the recombinant product. This finding argued for the need of physiological characterization of each recombinant pET-based *E. coli* BL21(DE3) strain in order to optimize recombinant protein production as well as to avoid sugar accumulation and resulting osmotic stress for the cells. Driven by this need we investigated different dynamic strategies to accelerate the establishment of the mechanistic model. In fact, we succeeded and propose a strategy comprising only three cultivations, including a batch, a standard fed-batch and a dynamic fed-batch cultivation, to determine the mechanistic q_s,glu_/q_s,lac,max_-model for pET-based *E. coli* BL21(DE3) strains. We believe that all scientists working with recombinant *E. coli* BL21(DE3) strains carrying the pET expression system will benefit from this study as we do not only present mechanistic platform knowledge, but also offer a strategy for fast determination of this mechanistic correlation.

## Results and Discussion

### Does the mechanistic q_s,glu_/q_s,lac,max_ model represent platform knowledge for *E. coli* BL21(DE3)?

The main motivation for this study was to test if the previously generated mechanistic model of q_s,glu_ and q_s,lac,max_ (Equation 1) for a recombinant *E. coli* BL21(DE3) strain producing a scFv with a pET expression system describes platform knowledge for pET-based *E. coli* BL21(DE3) strains.


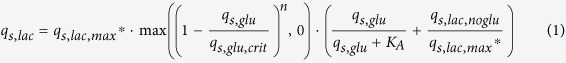


q_s,lac_ Specific lactose uptake rate [g/g/h].

q_s,lac,max_^*^ Maximum specific lactose uptake rate [g/g/h].

q_s,glu_ Specific glucose uptake rate [g/g/h].

q_s,glu,crit_ Critical specific glucose uptake rate up to which lactose is consumed [g/g/h].

q_s,lac,noglu_ Specific lactose uptake rate at q_s,glu_ = 0 [g/g/h].

K_A_ Affinity constant for the specific lactose uptake rate [g/g/h].

n Type of inhibition (noncompetitive, uncompetitive, competitive).

**Figure 1:**
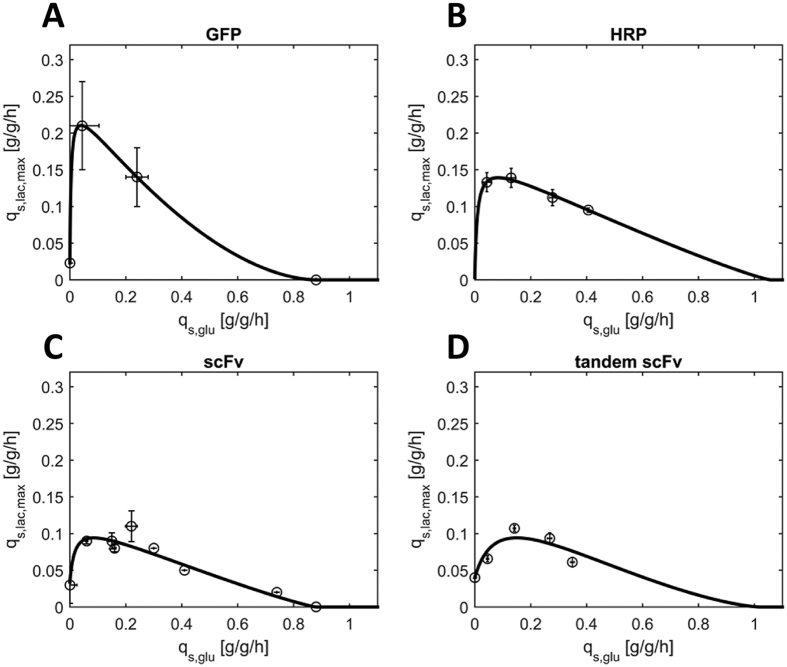
q_s,glu_/q_s,lac,max_-correlation for recombinant pET-based *E. coli* BL21(DE3) strains producing either (**A**) GFP, (**B**) HRP, (**C**) the scFv or (**D**) the tandem scFv. Data-points were obtained from batch and fed-batch cultivations with constant q_s,glu_ and excess lactose (“static experiments”) and subsequently fitted to the mechanistic model (Equation (1)).

Thus, we investigated this mechanistic correlation for four different pET-based recombinant *E. coli* BL21(DE3) strains producing either (1) the model protein green fluorescent protein (GFP), (2) the plant enzyme horseradish peroxidase (HRP; e.g. ref. 21), (3) a scFv against celiac disease^22^ and (4) a novel tandem construct of this scFv. HRP was produced in the periplasm of *E. coli*, whereas the other three proteins were produced in the cytoplasm. As suggested previously^16^, we performed at least four bioreactor cultivations, where we adjusted constant process parameters (hereafter referred to as “static experiments”), for each strain to determine the mechanistic correlation of q_s,glu_ and q_s,lac,max_. Then, we fitted the parameters of the mechanistic model (Equation (1))^16^ to the data. In fact, we were able to demonstrate that the mechanistic model was applicable for all four strains (Fig. 1). All curves followed the same trend: little lactose uptake at low q_s,glu_, followed by a steep increase in q_s,lac,max_, preceded by a comparatively shallow decrease at higher levels of q_s,glu_. We explain this trend as follows: At low levels of q_s,glu_ little lactose is taken up by the cells, as energy is required for the ATP-related lactose transport into the cell^23^. Towards higher q_s,glu_, q_s,lac,max_ increases but then gradually drops again due to the well-studied effects of carbon catabolite repression^16^,^18^,^19^,^20^,^24^. Due to the results depicted in Fig. 1, we concluded that the mechanistic model (Equation (1)) in fact describes platform knowledge for pET-based recombinant *E. coli* BL21(DE3) strains.

Even though all model parameters were within a physiologically meaningful range, we found striking differences between the four different strains (Table 1).

Thus, we performed an identifiability analysis to verify the model parameters. For the strains producing HRP, IGY and the scFv the analysis revealed identifiable parameters. However, for the strain producing the tandem scFv we had to include general knowledge derived from the other strains to identify the parameters making them slightly more error prone. We believe that for that strain the static experiments were not ideally distributed over the whole q_s,glu_ range (Fig. 1D). However, the normalized root mean square error (NRMSE) of all curves was below 10% attesting a good correlation between experimental data and data points from the fitted curves. Since all these strains were pET-based BL21(DE3) chassis strains, we concluded that mechanistic model parameters mainly depended on the recombinant product. This fact strongly argues for physiological strain characterization of each recombinant *E. coli* BL21(DE3) strain, not only to optimize recombinant protein production, but also to avoid sugar accumulation and thus osmotic stress for the *E. coli* cells^25^. In our previous study we proposed to conduct at least four bioreactor cultivations to determine the mechanistic q_s,glu_/q_s.lac,max_-correlation for any pET-based *E. coli* BL21(DE3) strain. Although this hypothesis obviously held true (Fig. 1), we were eager to find another strategy allowing faster strain characterization and thus faster bioprocess development.

### Use of dynamics for strain characterization

**Figure 2:**
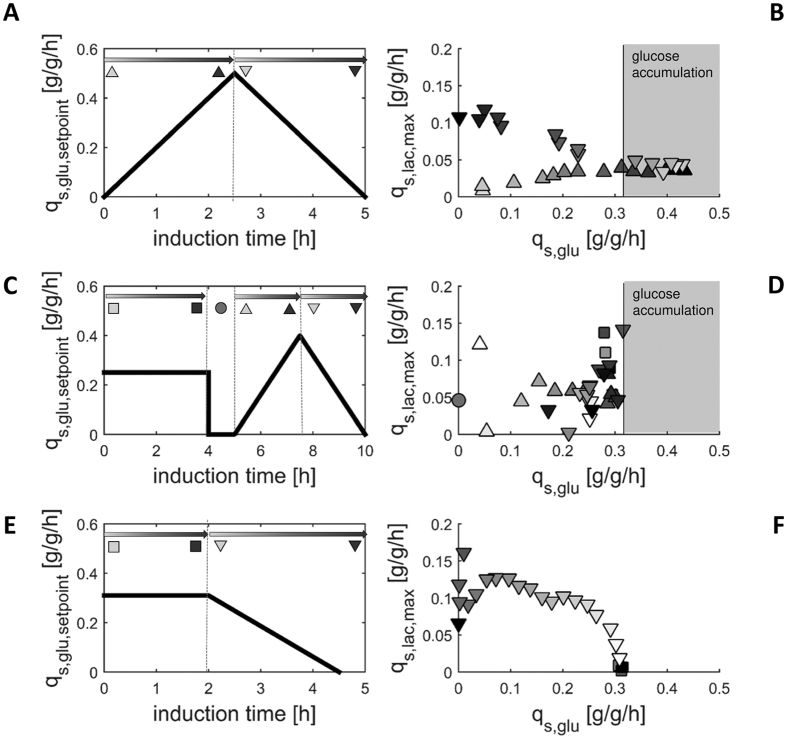
Dynamic bioprocess strategies (**A**,**C**,**E**) and respective resulting q_s,glu_/q_s,lac,max_-correlations (**B**,**D**,**F**) for the pET-based recombinant *E. coli* BL21(DE3) strain producing the tandem scFv against celiac disease. Different feeding phases are marked by different symbols. Time courses within phases go from light grey to dark grey, as indicated on the left. The shape and colour of the symbols of the q_s,glu_/q_s,lac,max_ data points on the right (**B**,**D**,**F**) correspond to the symbols on the left (**A**,**C**,**E**). Samples were taken every hour during adaptation, every 10 min during q_s,glu_ ramps and every hour after the ramp. For clear data representation error bars were omitted. The error was always between 7% and 15%.

**Figure 3:**
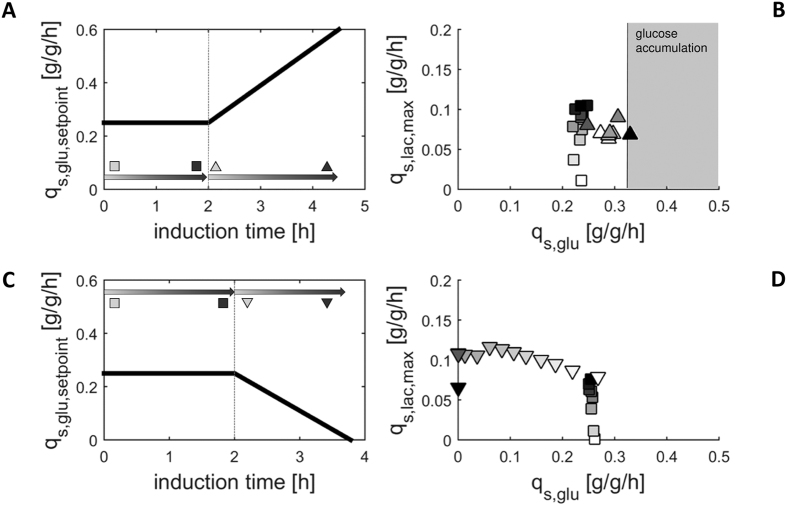
Optimized adaption followed by two dynamic experiments: ramp up (**A**,**B**) and ramp down (**C**,**D**). Samples were taken every 10 min during adaptation and every 30 min during q_s,glu_ ramps. In the ramp down experiment another sample was taken after 4 hours at q_s,glu_ = 0 g/g/h (black triangle in Fig. 3D). For clear data representation error bars were omitted. The error was always between 7% and 15%.

We used the pET-based recombinant *E. coli* BL21(DE3) strain producing the tandem scFv against celiac disease to test different dynamic methods to possibly allow faster determination of the q_s,glu_/q_s.lac,max_-correlation. Applying dynamic process conditions to accelerate bioprocess development is a common approach in our working group^26^,^27^,^28^,^29^,^30^. The different dynamic strategies tested are schematically depicted in Figs 2 and 3. In general, all experiments were conducted by employment of q_s,glu_-ramps and lactose in excess.

#### Hysteresis of q_s,glu_

In the first dynamic experiment q_s,glu_ was increased from 0 g/g/h to 0.5 g/g/h and then decreased again to 0 g/g/h within 5 h resulting in a hysteresis of the specific uptake rate of glucose (Fig. 2A). As shown in Fig. 2B, q_s,lac,max_ increased with increasing q_s,glu_. However, the absolute values were far lower compared to the results in static experiments (Fig. 1D). We hypothesized that *E. coli* needs time to adapt to lactose since enzymes required for uptake and metabolism of the disaccharide have to be expressed, a phenomenon which has been described for diauxic growth before^31^,^32^. Thus, we decided to include an adaptation phase to lactose before the q_s,glu_ ramp.

#### Adaptation followed by hysteresis of q_s,glu_

In the second dynamic experiment, we adjusted q_s,glu_ at 0.25 g/g/h for 4 h in the presence of lactose, followed by 1 h without glucose-feeding to investigate lactose uptake in the absence of glucose. Then, we again performed a q_s,glu_ hysteresis experiment (Fig. 2C). However, as shown in Fig. 2D the q_s,glu_/q_s,lac,max_-values did not follow the expected trend, but resulted in a quite chaotic cloud of q_s,lac,max_ data points. Viability measurements using FACS revealed fluctuating viability and up to 10% dead *E. coli* cells during this dynamic cultivation (Supplementary Figure S1). We hypothesized that 1 h without glucose feed and the long overall induction time of 10 h caused cell death and lysis which in turn led to fluctuating q_s_-values during the experiment. However, we found that q_s,lac,max_ values remained constant after approximately 2.0 h at q_s,glu_ = 0.25 g/g/h indicating that the cells were fully adapted (Supplementary Figure S2). Furthermore, we observed glucose accumulation at q_s,glu_ values higher than 0.32 g/g/h.

#### Adaptation followed by q_s,glu_ ramp down

Based on our observations that (1) adaption to lactose took approximately 2.0 h at q_s,glu_ 0.25 g/g/h, (2) glucose accumulation was observed for fully adapted cells at q_s,glu_ higher than 0.32 g/g/h, and (3) overall induction time should be kept short to maintain cell fitness (Supplementary Figure S1), we designed the third dynamic experiment as follows: the adaption phase was conducted for 2 h at q_s,glu_ of 0.31 g/g/h, before q_s,glu_ was ramped down to 0 g/g/h within 2.5 h (Fig. 2E). As shown in Fig. 2F, the data followed the expected trend (Fig. 1D) with two exceptions. First, q_s,lac,max_ values at q_s,glu_ of 0.31 g/g/h (light grey triangles in Fig. 2E) were much lower compared to the values obtained in static experiments (Fig. 1D). We concluded that 2 h of lactose presence at q_s,glu_ of 0.31 g/g/h were not sufficient for full adaptation and that adaptation at q_s,glu_ = 0.25 g/g/h was preferred. Furthermore, we observed quite high values for q_s,lac,max_ at low q_s,glu_ values. While in static experiments we determined a q_s,lac,max_ of 0.04 g/g/h at q_s,glu_ = 0 g/g/h (Fig. 1D, Table 1), we found q_s,lac,max_ values higher than 0.1 g/g/h in the dynamic experiment. However, we analyzed q_s,lac,max_ values for a prolonged time and interestingly observed a constant decrease of q_s,lac,max_ over time (Fig. 2F). We believe that the *E. coli* cells still harboured a great amount of enzymes required for the transport and metabolism of lactose once they had been cultivated in the dynamic ramp experiment and that the presence of these enzymes was only slowly reduced resulting in the initially high q_s,lac,max_ values. In contrast, when performing a static fed-batch experiment at q_s,glu_ of 0 g/g/h, the cells actually did not have the required energy to produce a great amount of these enzymes resulting in a lower q_s,lac,max_ (Fig. 1D). We obviously have to deal with great time effects when working with the pET-based *E. coli* systems and lactose induction^31^,^32^.

#### Optimized adaption followed by two ramp experiments

Based on the conclusions drawn from the first three dynamic experiments, we finally tested a strategy comprising two ramp experiments. To guarantee fast adaptation, we adapted the cells at q_s,glu_ = 0.25 g/g/h for 2.0 h. Once cells were fully adapted to lactose, indicated by a constant q_s,lac,max_, q_s,glu_ was either ramped up until glucose accumulation was observed (Fig. 3A) or ramped down to q_s,glu_ = 0 g/g/h at a rate of 0.14 g/g/h^2^ to guarantee a total induction time of less than 3 h (Fig. 3C).

**Figure 4:**
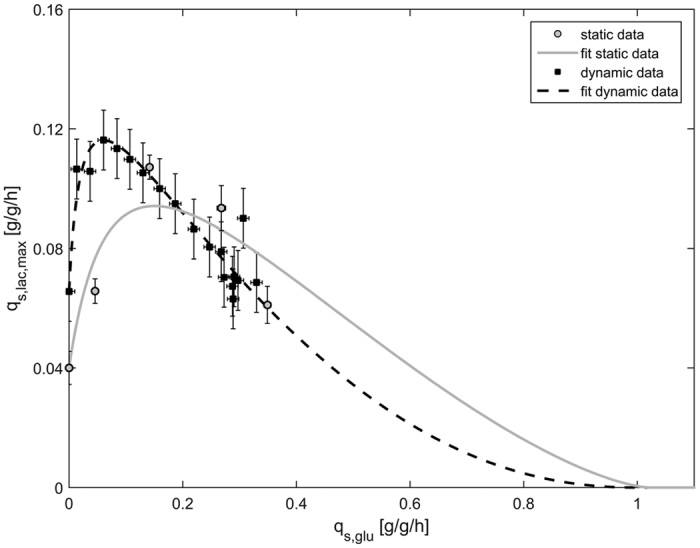
q_s,glu_/q_s,lac,max_-correlation derived from data points from static experiments (grey circles, solid line) and from dynamic ramp experiments (black squares, dashed line).

As shown in Fig. 3B, cells were fully adapted after 2.0 h. We observed a decrease of q_s,lac, max_ once we increased q_s,glu_, which is in line with our previous results (Fig. 1D). At q_s,glu_ = 0.32 g/g/h we again observed glucose accumulation confirming our previous observations. When we decreased q_s,glu_ from 0.25 g/g/h to 0 g/g/h we obtained q_s,lac,max_ values which followed the expected trend, but again were higher compared to the values from static experiments (Fig. 1D). We fitted the data of these two ramp experiments to the model (Equation (1)) and compared the model fit and the parameters to the results from static experiments (Fig. 4; Table 2).

**Figure 5:**
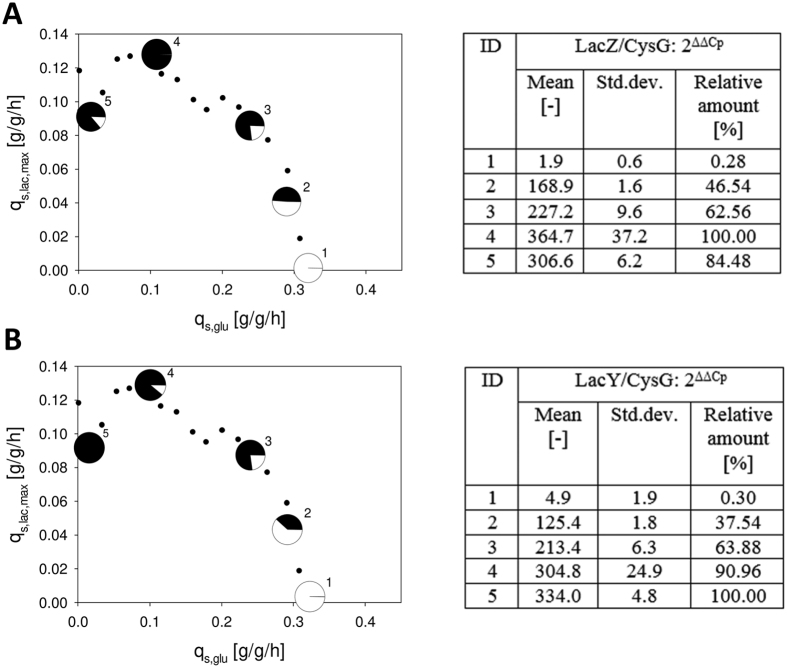
Time course of transcription of *lacZ* (**A**) and *lacY* (**B**). qPCR data were referenced to *CysG*. 2^ΔΔCp^ values were calculated by relating ΔCp to a reference sample which was taken before induction. Pie charts display the percentage of transcription in relation to the highest 2^ΔΔCp^ value obtained.

As shown in Fig. 4, the two model fits were very different: the main discrepancy between the two model fits was found at low q_s,glu_ values. However, this also impacted the shape of the curve at high q_s,glu_ values. The NRMSE of the curve derived from ramp experiments compared to the data derived from static experiments was 22.1% (Table 2). Again, we hypothesized that there was still a high amount of enzymes for lactose uptake and metabolism available in the cells distorting the true q_s,glu_/q_s,lac,max_-correlation at low q_s,glu_ setpoints. Apparently, the ramp speed was higher than the physiological adaptation of the cells. To put this hypothesis to test and further confirm our observations of the q_s,glu_/q_s,lac,max_-correlation by molecular biological data, we performed qPCR-analysis of β-Galactosidase (LacZ) and β-Galactosid-Permease (LacY) (Fig. 5). The transcription of both genes was regulated by the well-studied lac operon which is why abundance of mRNA of either gene depended on the availability of inducer^33^.

As shown in Fig. 5, transcription levels of both genes strongly correlated with q_s,lac,max_. Transcription levels gradually increased with decreasing q_s,glu_ and the consequent increase in q_s,lac,max_. The transcription level of *lacZ* was highest at q_s,glu_ = 0.11 g/g/h and then decreased at q_s,glu_ = 0 g/g/h, where also q_s,lac,max_ decreased. However, the transcription level of *lacY* was highest at q_s,glu_ = 0 g/g/h. We have no explanation for this difference in abundance of *lacY* and *lacZ* at this point. However, comparing the transcript levels of both genes at q_s,glu_ = 0 g/g/h and q_s,glu_ = 0.24 g/g/h, where approximately the same q_s,lac,max_ was reached and thus the same amount of inducer was present, we detected large discrepancies (Fig. 5). The amount of mRNA for both genes was 20–40% higher at q_s,glu_ = 0 g/g/h, supporting our hypothesis that the q_s,glu_ ramp was faster than the adaptation capacity of the cells. Thus, we concluded that only static experiments reveal the true q_s,glu_/q_s,lac,max_ at low q_s,glu_ levels.

### Proposed experimental strategy to determine the mechanistic q_s,glu_/q_s,lac,max_ correlation

In order to find a fast experimental strategy to determine the q_s,glu_/q_s,lac,max_-correlation for pET-based recombinant *E. coli* BL21(DE3) strains, we differently combined static and dynamic experiments and performed sensitivity analyses of the models to investigate the error of fit (Table 2).

**Figure 6:**
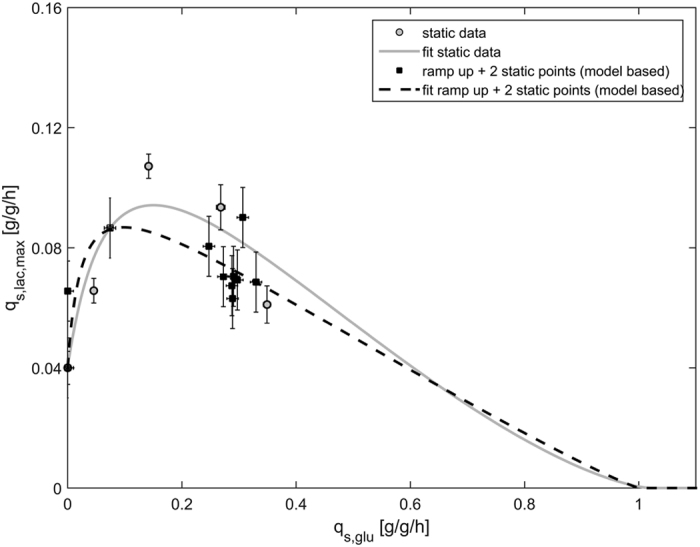
Comparison of the optimal fit for the static experiments and the combination of the dynamic ramp up and two static experiments derived from a model-based experimental design.

As shown in the values of NMRSE (Table 2), data derived from dynamic ramp experiments gave unsatisfactory model fits with respect to static data points (NMRSE = 22.1% and 17.9%, respectively). Combining the data from all static experiments and the ramp up experiment gave a satisfactory fit and a NMRSE of only 12.5% (Table 2). However, since we wanted to develop a strategy comprising less experiments, we conducted a model based experimental design by sensitivity analysis^34^: we combined the data from the ramp up experiment and from two static experiments at q_s,glu_ = 0 g/g/h and q_s,glu_ = 0.074 g/g/h, respectively, and fitted the model (Fig. 6).

In fact, this combination of the dynamic ramp up and two static experiments gave a satisfactory fit and a NMRSE of only 12.8% (Table 2). In terms of the deviating parameters K_A_ and q_s,lac,max_^*^ (Table 2), we had found that those parameters were hard to identify from static experiments before and were therefore less trustworthy (*vide supra*). In contrast we were able to identify these parameters from the combination of the dynamic and two static experiments when we performed a practical identifiability analysis. We concluded that the parameters derived from this experimental combination are in fact more trustworthy than the parameters derived from static experiments only. To prove the applicability of the model, we assumed different q_s,glu_ values between 0.1 and 0.8 g/g/h and calculated the respective q_s,lac,max_ values using the model derived from static experiments only as well as from the combination of the dynamic and two static experiments (Table 3).

As shown in Table 3, the deviation between the calculated values for q_s,lac,max_ for both models were below 15%. Furthermore, a direct comparison of the two model fits gave a NRMSE of only 5.90%, confirming the high similarity thereof. Thus, we propose a strategy comprising only three experiments to determine the mechanistic q_s,glu_/q_s,lac,max_ correlation for a pET-based recombinant *E. coli* BL21(DE3) strain. Our strategy can be summarized as:

Perform a batch cultivation on glucose, followed by a lactose pulse to determine the parameter q_s,lac,noglu_ (static experiment No. 1).

Perform a batch cultivation on glucose, followed by a fed-batch cultivation with lactose in excess (>5 g/L) at a low q_s,glu_ value of around 0.1 g/g/h, as sensitivity analysis found data points in this region to be crucial for correct parameter estimation (static experiment No. 2).

Perform a batch cultivation on glucose, followed by a dynamic experiment: Adapt *E. coli* to lactose at intermediate q_s,glu_ of around 0.25 g/g/h for 2.0 h. However, since adaptation time might differ from strain to strain we recommend at-line HPLC measurements of sugar concentrations and biomass-estimation by OD_600_ every 30 min to reliably determine full adaptation to lactose (Supplementary Figure S3). After adaption, linearly increase q_s,glu_ at a rate of 0.14 g/g/h^2^ to guarantee a total induction duration of less than 5 h in order to maintain cell fitness (Supplementary Figure S1).

Plot the q_s,glu_/q_s,lac,max_ values and fit them to the mechanistic equation to be able to determine the model parameters and thus the physiological limits of the respective *E. coli* BL21(DE3) strain.

## Conclusions

In this study we were able to show that our previously generated mechanistic q_s,glu_/q_s,lac,max_ model in fact describes platform knowledge for pET-based recombinant *E. coli* BL21(DE3) strains. We found that model parameters were greatly affected by the recombinant product, pushing for physiological strain characterization of each *E. coli* strain to allow efficient recombinant protein production and to avoid sugar accumulation and the resulting osmotic stress for the cells. We compared data from different dynamic strategies to the data obtained from static experiments. Finally, we propose a strategy comprising only one batch cultivation, one fed-batch cultivation as well as one dynamic experiment, to reliably determine the mechanistic model for q_s,glu_/q_s,lac,max_ and get trustworthy model parameters for pET-based recombinant *E. coli* BL21(DE3) strains.

## Material and Methods

### Strains

All cultivations were conducted with the *E. coli* BL21(DE3) strain (Life technologies, Carlsbad, CA, USA). Green fluorescent protein (GFP) was expressed using a pET21a(+) plasmid. Periplasmic horseradish peroxidase (HRP) was expressed from a pET39(+) plasmid. For expression of both the recombinant scFv and the tandem-scFv a pET28a(+) plasmid was used.

### Bioreactor cultivations

#### Media

All fermentations were carried out in defined minimal medium according to DeLisa *et al*.^35^. Depending on the antibiotic resistance genes on the plasmid the medium was either supplemented with 0.1 g/L ampicillin or 0.02 g/L kanamycin. Feeds contained 250 g/L Glucose or 200 g/L Lactose, respectively.

#### Pre-culture

Pre-cultures were conducted by inoculating 500 mL of sterile DeLisa pre-culture medium in a 2500 mL High-Yield shake flask with frozen stocks (1.5 mL, −80 °C) and subsequent incubation in an Infors HR Multitron shaker (Infors, Bottmingen, Switzerland) at 37 °C and 230 rpm for 20 h. Bioreactors were inoculated using a tenth of the final batch volume.

#### Overall cultivation strategy

All cultivations comprised three phases (batch, non-induced fed-batch, induced fed-batch) including dynamic experiments during induction. Induction was performed by lactose which was applied by a pulse to reach concentrations of 20–25 g/L and then always kept higher than 5 g/L. For that purpose at-line lactose measurements by HPLC were performed.

#### Static experiments

Experiments for static strain characterisation were performed in DASbox Mini Bioreactors (Eppendorf, Hamburg, Germany) with a working volume of 250 mL. The reactors were supplied with 2 vvm of a mixture of pressurized air and oxygen, the ratio was adjusted in a way to keep dissolved oxygen (dO) above 40% during cultivation. dO was monitored using a fluorescence dissolved oxygen electrode Visiferm DO120 (Hamilton, Reno, NV, USA). The reactors were stirred constantly at 2,000 rpm. pH was monitored by a pH-Sensor EasyFerm Plus (Hamilton, Reno, NV, USA), and kept at 7.2. If necessary, it was adjusted by addition of NH_4_OH (12.5%). Base uptake was monitored via flowrates with the DASbox MP8 Multipumpmodul. A DASGIP GA gas analyzer (Eppendorf, Hamburg, Germany) was used for monitoring CO_2_ and O_2_ concentrations in the offgas. All process parameters were logged and controlled by DASware control.

The batch phase was carried out at 35 °C and yielded a biomass concentration of 8–9 g dry cell weight (DCW) per liter. When the CO_2_ off-gas signal dropped, indicating the end of the batch phase or glucose depletion respectively, a fed-batch to generate biomass was conducted. Fed-batch phases were conducted at a q_s,glu_ of 0.25 g/g/h. When the DCW reached 25 g/L, the temperature was set to 30 °C and cultures were induced by a lactose pulse to reach a lactose concentration of 25 g/L in the bioreactor. The feed rate was adjusted to control q_s,glu_ (Equation (2)). DCW in the bioreactor was estimated by using a Soft-sensor-tool^36^.


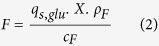


F feedrate [g/h].

q_s,glu_ specific glucose uptake rate [g/g/h].

X absolute biomass [g].

ρ_F_ feed density [g/L].

c_F_ feed concentration [g/L].

#### Dynamic experiments

For development of the dynamic strategy, batch and fed-batch cultivations were done in a stainless steel Sartorius Biostat Cplus bioreactor (Sartorius, Göttingen, Germany) with a working volume of 10 L. The reactor was stirred at 1,400 rpm. pH was monitored with an EasyFerm electrode (Hamilton, Reno, NV, USA) and was kept at 7.2 by addition of NH_4_OH (12.5%) or HCl (18.75%), respectively. Base and acid consumption were determined gravimetrically. For monitoring O_2_ and CO_2_ concentrations in the offgas a DASGIP GA gas analyzer (Eppendorf, Hamburg, Germany) was used. Aeration was performed with a mixture of pressurized air and pure oxygen at 1.5 vvm, varying the ratio of pressurized air to pure oxygen in a way that dissolved oxygen (dO) was kept above 40% throughout all cultivations. dO was monitored with a fluorescence dissolved oxygen electrode Visiferm DO120 (Hamilton, Reno, NV, USA). Process parameters were logged and controlled by the process information management system Lucullus (Biospectra, Schlieren, Switzerland).

The batch phase was conducted at 35 °C with an initial glucose concentration of 20 g/L and led to biomass concentrations of 8–9 g DCW per litre. After the end of the batch phase, a fed-batch to generate biomass was carried out.

We fed at a constant specific glucose uptake rate (q_s,glu_) of 0.25 g/g/h. When the DCW reached 25 g/L, the temperature was lowered to 30 °C and the culture was induced by lactose. During non-induced fed-batch as well as during induction with lactose, DCW was calculated assuming a constant biomass yield (Y_X/S_ = 0.37 g/g, own unpublished data; Equation (3)). The feed rate was adjusted to control q_s,glu_ and was calculated according to Equation (2).


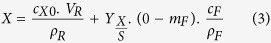


c_X0_ initial biomass concentration [g/L].

V_R_ reactor volume [L].

ρ_R_ density of fermentation broth [g/L].

Y_X/S_ biomass-yield [g/g].

m_F_ balance signal of feed balance [g].

ρ_F_ feed density [g/L].

c_F_ feed concentration [g/L].

#### Sampling and Analysis

Samples were taken at the beginning and end of the batch and the non-induced fed-batch. During induction, sampling was performed every 30 min to analyze DCW and OD_600_. In addition, every 10 min supernatant for sugar analysis was collected via an in-line ceramic 0.2 μm filtration probe (IBA, Heiligenstadt, Germany). DCW was determined by centrifuging (4500 g, 4 °C, 10 min) 1 mL cultivation broth, washing the obtained cell pellet with a 0.9% NaCl solution and subsequent drying at 105 °C for 72 h. Optical density at 600 nm (OD_600_) was determined using a Genesys 20 photometer (Thermo Scientific,Waltham, MA, USA). For staying within the linear range of the photometer (OD_600_ 0.1–0.8) samples were diluted with 0.9% NaCl solution. A calibration correlation OD_600_ to DCW was established. Sugar concentrations were analysed via HPLC (Thermo Scientific, Waltham, MA, USA) on a Supelcogel column (Supelco Inc., Bellefonte, PA, USA) with 0.1% H_3_PO_4_ as eluent at a constant flow of 0.5 ml/min. The method for sugar analysis lasted 15 min. Analysis of the chromatograms was performed using Chromeleon Software (Dionex, Sunnyvale, CA, USA). In order to examine cell-death during bioreactor cultivations, fluorescence-activated cell sorting (FACS) was conducted via Cube 8 (Sysmex Partec, Görlitz, Germany) according to Langemann *et al*.^37^.

### Data-Analysis

Fitting the data to the mechanistic q_s,glu_/q_s,lac,max_-model was carried out according to our previous study^16^. In short, unknown parameters of Equation (1) were identified using the Nelder-Mead simplex method in MATLAB R2014b to minimize the objective function with respect to physiologically meaningful boundaries (Equation (4)).


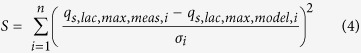


S objective function.

q_s,lac,max,meas,i_ i^th^ measurement of q_s,lac,max_.

q_s,lac,max,model,i_ predicted q_s,lac,max_ at timepoint of i^th^ measurement.

σ_i_ standard deviation of the i^th^ data point.

For calculating q_s,lac,max_-values biomass concentrations as well as lactose amounts were interpolated using a Savitzky-Golay-filter in MATLAB R2014b. A practical parameter identifiability analysis was performed by a method similar to Raue^38^: for each parameter a physiologically meaningful range [p_min_, p_max_] was defined and the parameter was held fix at various values inside this range. The objective function S (Equation (4)) was then iteratively minimized for each parameter value with respect to the other parameters resulting in a trajectory S_p_. If S_p_ has a minimum the parameter can be interpreted to be identifiable.

To suggest a model based experimental design to optimally estimate the mechanistic model parameters a local sensitivity analysis of the model parameters was conducted similar to Franceschini *et al*.^34^: the parameters were disturbed by ±10%. Those q_s,glu_ values where the deviation of the q_s,lac_ values is maximal with respect to the original parameter values were assumed to contain maximal information to estimate the parameter.

### qPCR-Analysis

qPCR-Analysis was done as a commercial service offered by Microsynth AG (Balgach, Switzerland). Reference gene *CysG* was used. 2^ΔΔCp^-values were calculated and normalized to the highest value found.

## Additional Information

**How to cite this article:** Wurm, D. J. *et al*. Mechanistic platform knowledge of concomitant sugar uptake in *Escherichia coli* BL21(DE3) strains. *Sci. Rep.* **7**, 45072; doi: 10.1038/srep45072 (2017).

**Publisher's note:** Springer Nature remains neutral with regard to jurisdictional claims in published maps and institutional affiliations.

## Supplementary Material

Supplementary Information

## Figures and Tables

**Table 1:** Model parameters for recombinant pET-based *E. coli* BL21(DE3) strains producing GFP, HRP, the scFv or the tadem scFv.

rec. product	q_s,lac,max_^*^ [g/g/h]	K_A_ [g/g/h]	q_s,glu,crit_ [g/g/h]	n [-]	q_s,lac,noglu_ [g/g/h]	NRMSE [%]
GFP	0.23	0.0042	0.88	1.77	0.023	0.04
HRP	0.17	0.0092	1.06	1.15	0.0032	5.14
scFv	0.09	0.019	0.88	1.16	0.034	9.72
tandem scFv	0.13	0.094	1.02	1.48	0.040	9.11

The mechanistic model is shown in Equation (1).

**Table 2:** Comparison of parameters and NRMSE of models fitted to data from static and dynamic experiments as well as combinations thereof.

Datasets	q_s,lac,max_^*^ [g/g/h]	K_A_ [g/g/h]	q_s,glu,crit_ [g/g/h]	n [-]	q_s,lac,noglu_ [g/g/h]	NRMSE [%]
Static data	0.13	0.094	1.02	1.48	0.040	9.11
Dynamic data (ramp up and down)	0.093	0.023	1.00	2.17	0.066	22.1
Dynamic data (ramp up)	0.072	0.392	1.00	0.83	0.066	17.9
Dynamic data (ramp up) & all static data	0.075	0.025	1.00	1.14	0.040	12.5
Dynamic data (ramp up) & two static data points (model based)	0.072	0.025	1.00	1.11	0.040	12.8

**Table 3:** Comparison of q_s,lac,max_ values at different q_s,glu_ setpoints calculated from models derived from static experiments only as well as from the combination of the dynamic and two static experiments.

q_s,glu_ [g/g/h]	0.1	0.2	0.3	0.4	0.5	0.6	0.7	0.8
q_s,lac,max_ [g/g/h] static fit = A	0.091	0.092	0.082	0.069	0.055	0.041	0.028	0.016
q_s,lac,max_ [g/g/h] combination fit = B	0.087	0.081	0.072	0.061	0.050	0.039	0.029	0.018
percentual deviation = (A-B)/A	4.9%	12.2%	13.2%	11.6%	8.3%	3.4%	−3.7%	−13.8%
