# Supplementary material for: Mechanistic platform knowledge of concomitant sugar uptake in Escherichia coli BL21(DE3) strains
Source: Sci Rep. 2017 Mar 23;7:45072. doi: 10.1038/srep45072 (PMC5362885; doi:10.1038/srep45072)
Supplement: Supplementary Information [file srep45072-s1.pdf]

Mechanistic platform knowledge of concomitant sugar uptake in *Escherichia coli* BL21(DE3) strains

Supplementary Files

David J. Wurm[1*], Johanna Hausjell[1*], Sophia Ulonska[1], Christoph Herwig[1,2], Oliver Spadiut[1,2]

[1]*Research Division Biochemical Engineering, Institute of Chemical Engineering, Vienna University of Technology, Vienna, Austria*

[2]*Christian Doppler Laboratory for Mechanistic and Physiological Methods for Improved Bioprocesses, Institute of Chemical Engineering, Vienna University of Technology, Vienna, Austria*

**These authors contributed equally to this work*

Correspondence and requests for materials should be addressed to:

Oliver Spadiut, TU Wien, Institute of Chemical Engineering, Research Area Biochemical Engineering, Gumpendorfer Strasse 1a, 1060 Vienna, Austria. Tel: +43 1 58801 166473, Fax: +43 1 58801 166980, Email: oliver.spadiut@tuwien.ac.at

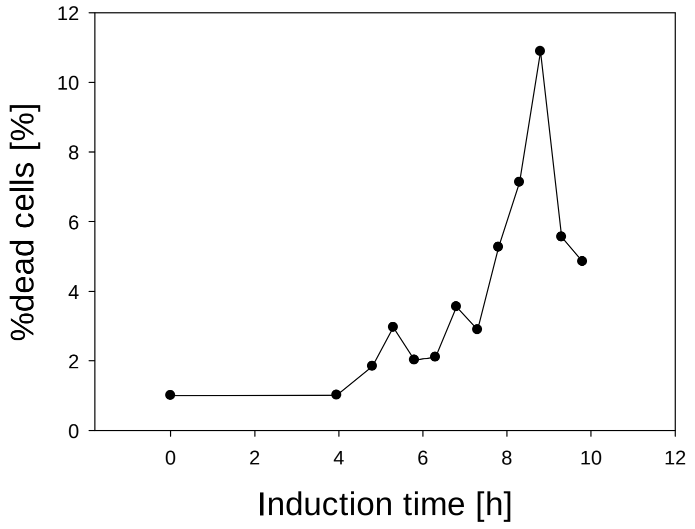

Supplementary Fig. 1: Dead cells during cultivation shown in Figure 2C and 2D measured by Flow cytometry.

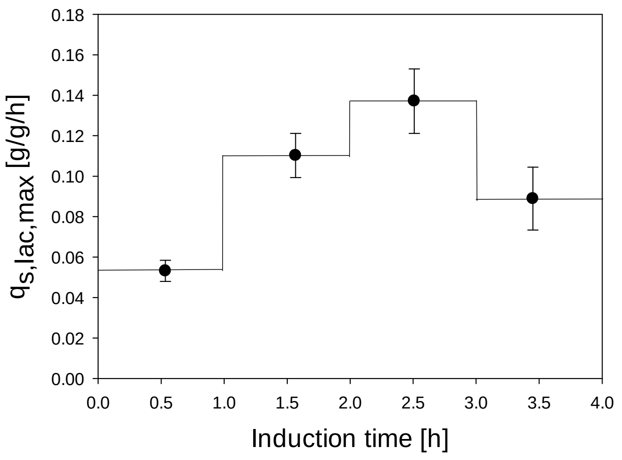

Supplementary Fig. 2: $q_{s,lac,max}$ of cultivation shown in Figure 2C and 2D values during first 4 h after induction for investigating duration of adaption phase. Errors were calculated by error propagation from standard deviations of DCW and sugar measurements which were carried out in triplicates.

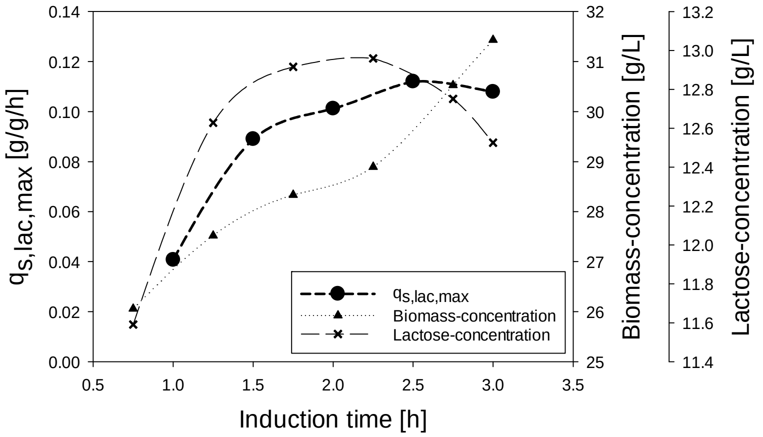

Supplementary Fig. 3: Exemplary data for obtained $q_{s,lac,max}$ values from at-line determination of biomass (via OD_{600}) and sugar analysis in the supernatant (via HPLC) showing a constant $q_{s,lac}$ and thus full adaption of the cells after 2 h.
